# Effect of Rosemary Cream on Episiotomy Wound Healing in Primiparous Women: A Randomized Clinical Trial

**DOI:** 10.1186/s12906-022-03675-1

**Published:** 2022-08-26

**Authors:** Fatemeh Hadizadeh-Talasaz, Fariba Mardani, Narjes Bahri, Hassan Rakhshandeh, Nasim Khajavian, Marzieh Taghieh

**Affiliations:** 1 Department of Midwifery, Faculty of Medicine, Social Development & Health Promotion Research Center, Gonabad University of Medical Sciences, Gonabad, Iran; 2 Department of Midwifery, Marvdasht Shahid Motahari Hospital, Shiraz University of Medical Sciences, Shiraz, Iran; 3 Department of Midwifery, Faculty of Medicine, Social Determinants of Health Research Center, Gonabad University of Medical Sciences, Gonabad, Iran; 4 Pharmacological Research Center of Medicinal Plants, Mashhad University of Medical Sciences, Mashhad, Iran; 5 Department of Pharmacology, Faculty of Medicine, Mashhad University of Medical Sciences, Mashhad, Iran; 6 Department of Epidemiology and Biostatistics, School of Health, Social Determinants of Health Research Center, Gonabad University of Medical Sciences, Gonabad, Iran; 7 Department of Obstetrics and Gynecology, Marvdasht Shahid Motahari Hospital, Shiraz University of Medical Sciences, Shiraz, Iran

**Keywords:** Episiotomy, Primiparous, Rosemary, Wound healing

## Abstract

**Background:**

Delay in episiotomy wound healing can lead to infection. The application of natural antimicrobial agents isolated from herbal essences can be a good strategy to prevent the growth of bacteria and promote the wound healing process. Therefore, this study aimed to determine the effect of rosemary cream on episiotomy wound healing in primiparous women.

**Methods:**

This triple-blind randomized clinical trial was conducted on 80 primiparous pregnant women who were referred to the maternity ward of Shahid Motahari Hospital in Marvdasht, Iran, from September 2019 to March 2020. These women were randomly assigned into two groups of intervention (rosemary cream) and control (placebo), using variable quadruple blocks. Both groups applied the prescribed cream (in a dose of 2 cm) uniformly on the sutured area to cover it with the cream. The cream was applied twice a day for 10 consecutive days postpartum, and the rate of wound healing was evaluated by the researcher in the first 12 h and at days 4 and 10 postpartum using the REEDA scale. The data were analyzed using SPSS software (Version 19) through the Chi-square test, Mann-Whitney U test, student’s t-test, and Fisher’s test. A *p*-value less than 0.05 (*P* < 0.05) was considered statistically significant.

**Results:**

The mean ± SD REEDA score on the fourth day postpartum was obtained at 3.82 ± 0.93 and 4.25 ± 1.29 in the groups of rosemary cream and placebo, respectively (*P* = 0.17). However, the mean ± SD REEDA scores on the 10th day postpartum were determined at 0.75 ± 0.74 and 3.32 ± 2.54 in the rosemary cream and placebo groups, respectively, indicating a higher rate of episiotomy wound healing in the group of rosemary cream compared to placebo (*P* < 0.001).

**Conclusion:**

Based on the obtained results, it seems that rosemary cream can be effective in healing episiotomy wounds in primiparous women. However, further studies are suggested to confirm the findings of this study.

**Trial registration:**

This trial was registered in the Iranian Registry of Clinical Trials in 24/08/2019 with the IRCT ID: IRCT20190308042971N1. The first participant enrolled in this trial in September 2019. URL of registry: https://en.irct.ir/trial/40092.

## Background

Episiotomy is a surgery conducted to enlarge the vaginal orifice by making an incision in the perineum during the late second stage of labor [1] to facilitate the expulsion of the fetus [2] and reduce grades 3 and 4 perineal tears occurring during the second stage of labor [3]. Although episiotomy is the most common surgical procedure in obstetrics [4], the use of this surgical method has decreased nowadays due to the lack of evidence on its protective effects against anal sphincter injuries caused by vaginal delivery [1]. The World Health Organization [WHO] has recommended episiotomy for less than 10% of vaginal deliveries and has limited its use to complicated vaginal deliveries, such as breech, shoulder dystocia, forceps/ vacuum delivery, fetal distress, wounds caused by female genital circumcision, and minor repair of anal sphincter injuries. However, the rate of episiotomy is still high in developing countries [5]. The rate of episiotomy was reported to be 41.5% in a study conducted in Shahroud, Iran, from 2014 to 2015 [6].

Episiotomy is a type of surgical incision that does not reduce the risk of severe perineal rupture, rather it increases the risk of such complications as perineal rupture, perineal pain, wound infection, and postpartum hemorrhage, and subsequent dyspareunia [5].

The risk of perineal ulcer infection is high due to the proximity of the episiotomy site to the vagina and rectum. Moreover, the fact that access to this area is difficult for the mother presents the need for undertaking a quick restorative procedure [7, 8]. It should be noted that delays in wound healing can increase the risk of infection (4, 8). Therefore, wound care during the postpartum period is of particular importance and can facilitate mothers’ return to normal life [4].

Wounds are common medical problems that disturb both patients and physicians who provide care to them. However, despite the extent of wounds, there are very few accepted treatments for improving the process of wound healing [9].

Topical antibiotics and antimicrobials can reduce the risk of infection during wound healing. Antibiotics have become increasingly ineffective in recent years due to the development of resistant strains [10, 11]. Therefore, the use of natural antimicrobial agents isolated from herbal essences is a good strategy to prevent bacterial growth and facilitate the process of wound healing [12]. The role of herbs in the wound healing process involves disinfection and the creation of the right condition for the natural healing process (13).

Iranians have a long history of medicinal plants use [13]. Women are major consumers of herbal medicinal products for health and treatment purposes and continue to apply herbal products even during pregnancy and after childbirth [14]. Numerous studies have been performed on episiotomy wound healing using different herbs, such as frankincense [15], green tea [16], *Myrtus communis* [17], *Pistacia atlantica* [18], *aloe vera* [19], honey, and curcumin [4].

Rosemary is a medicinal plant, native to the Mediterranean region, and is cultivated all over the world [20]. Based on the evidence, rosemary has antioxidant, anti-inflammatory, anti-viral, anti-fungal, and anti-bacterial effects [21] which can be effective in wound healing [22, 23]. Moreover, rosemary has antioxidant properties due to the presence of such compounds as carnosic acid, carnosol, rosemary acid, diterpene, triterpenoids, phenolic acid, and flavonoids [22]. The presence of these compounds in rosemary gives it an inhibitory power against the growth of pathogens, reduces inflammatory responses in the body, and protects living tissues [24].

Some studies have reported the positive effects of rosemary on wound healing. In a study performed by Bestagno et al. [2017] in Chile, a mouthwash containing rosemary extract was reported to heal oral mucosal wounds [25]. The results of a study performed by Khoshoei et al. [2021] in Rafsanjan, Iran, showed that rosemary ointment facilitated the healing process of grade 1 pressure ulcers in the intensive care unit (ICU) and prevented the progression of these ulcers [26]. The results of a study conducted by Araujo et al. (2017) in Brazil revealed that the topical application of rosemary essential oil was effective in healing skin surgical wounds in mice [27].

Regarding the side effects of synthetic drugs, the use of medicinal plants is a better option in wound healing due to minimal side effects and cost-effectiveness, compared to synthetic compounds [28]. The medicinal application of plants requires clinical evidence [3]; however, no study has yet examined the effect of rosemary on episiotomy wound healing. Therefore, this study aimed to evaluate the effect of rosemary cream on episiotomy wound healing in primiparous women.

## Methods

### Study design

This study is a randomized, triple-blind clinical trial with two parallel groups of intervention and placebo. The study protocol was approved by the Research Ethics Committee in Gonabad University of Medical Sciences, Gonabad, Iran (IR.GMU.REC.1397.089) and registered in the Iranian Clinical Trial Center on 24 August 2019 (Registration number: IRCT20190308042971N1; available at: https://en.irct.ir/trial/40092). This clinical trial has been reported based on the CONSORT 2010 checklist [29].

### Participants and setting

This study was conducted on primiparous women who were referred to Shahid Motahari Hospital in Marvdasht, Iran, from September 2019 to March 2020.

The inclusion criteria included primiparous mother, the age range of 18-35 years, single pregnancy with a cephalic presentation, gestational age of 37-42 weeks, infant weight of 2500-4000 g, mediolateral episiotomy, lack of clear symptoms of perineal and vaginal infections (odorous secretions and burning and itching sensation) at admission, the absence of anal and perineal lesions or the history of regenerative surgery on vagina and perineum, lack of severe cystocele and rectocele, lack of drug and tobacco use (cigarette and hookah) based on the mother’s comment, lack of diseases that disrupt wound healing (e.g., diabetes, severe anemia, immune system disorder), non-use of medications that affect wound healing, non-rupture of amniotic sac for more than 18 h, no history of instrumental delivery (i.e., forceps and vacuum delivery), lack of perineum rupture grade 3 and 4, lack of precipitous labor, lack of assisted placenta delivery (with hands), and the absence of neonatal abnormality.

The exclusion criteria included performing curettage in the first 24 h postpartum, the presence of abnormal postpartum hemorrhage, re-manipulation of the perineum after episiotomy repair, infant hospitalization, infant death, hematoma formation in the episiotomy site, improper application of the cream, sensitivity to rosemary cream, having sexual intercourse in the first 10 days postpartum, symptoms of infection in the episiotomy site, episiotomy dehiscence, consumption of drugs affecting wound healing during the study, the puerperal fever in the mother, unwillingness to continue participating in the study, and non-referral to the clinic on days 4 and 10 postpartum.

### Sample size

The sample size needed for comparing the two study groups was determined using G *Power software (Version 3.1.9.2) and according to a similar study conducted previously [30]. The effect size and the first type error in this study were set at 0.7 and 0.05, respectively. The smallest sample size was determined to be 72 regarding a test power of 90%. However, considering the attrition rate of 30%, the sample size was increased to 92, with 46 participants included in each study group.

### Randomization and blinding

Samples were randomly assigned into two groups of intervention and control using variable quadruple blocks. In total, six possible block arrangements were listed and one number from 1 to 6 was assigned to each block. One number (between 1 and 6) was then randomly selected and individuals were assigned to the groups (A) and (B), based on the respective block, and this was repeated until the sample size was completed. This sample allocation process was performed by a third party to prevent bias. Researchers, the study participants, and the statistical specialist who analyzed the data were blinded to group allocations. At the end of the study, blind codes were decoded and patients who were assigned to each group were identified.

### Study Instruments

Data collection tools included demographic characteristics form (including such items as maternal age, maternal education and occupation, husband’s education and occupation, and income level), midwifery and labor characteristics form (including such items as gestational age, duration of amniotic sac rupture, labor duration, baby weight, and baby head circumference), daily form of drug use registration, medication side effect form, rosemary cream use satisfaction form, and wound healing REEDA scale.

The wound healing REEDA scale is a tool developed by Davidson in 1974 to assess episiotomy wound healing through the evaluation of redness, edema, ecchymosis, discharge, and approximation of the wound edges [31]. This scale included five items and a score between 0 and 3 was assigned to each item. The total score of the scale was between 0 (maximum improvement) and 15 (minimum improvement). The total scores 0, between 1 and 5, between 6 and 10, and between 11 and 15 indicated recovery, moderate recovery, poor recovery, and lack of recovery, respectively [32]. The validity and reliability of this scale were confirmed using the content validity method and Cronbach’s alpha, according to previous studies [33].

### Interventions and outcomes

Rosemary cream and placebo cream were prepared by the Pharmacological Research Center of Medicinal Plants, Mashhad, Iran. Initially, 1000 g of rosemary plant (Rosmarinus Officials L.) were collected from the campus of Mashhad University of Medical Sciences, Iran. The plant’s identity was confirmed by the herbarium of the Faculty of Pharmacy, Mashhad University of Medical Sciences, Mashhad, Iran (Herbarium number: 132090). Afterward, the rosemary plant was first washed, dried, and crushed by an electric grinder to prepare rosemary alcoholic extract. The obtained powder was then extracted with 70% alcohol using a Soxhlet extractor. The concentrated extract was obtained in the pharmacology laboratory of the medical school after the complete removal of the solvent by the solvent removal device (rotary evaporator). Subsequently, the concentrated rosemary extract was mixed with Farabi base cream and 3% rosemary cream was prepared. The resulting mixture was poured into 30 g aluminum tubes and the tube was sealed after cooling. A placebo cream (30 g) was made by a pharmacist and contained a simple base cream and a few drops of standard edible brown dye. All steps of mixing the extract with the base cream and filling the tubes were performed under a laminar flow hood. Farabi base cream, which is available in the Iranian pharmaceutical market and is often used for making creams, has been prepared by Farabi Pharmaceutical Company in Iran. This base cream is standard and has a production license, and any researcher can purchase and use it. The ingredients of Farabi base cream include Stevastenaryl alcohol, petroleum gel, glycerin, mineral oil, preservative, and antioxidants (Table 1).

**Table 1:** Ingredients of rosemary cream and placebo

Ingredients of cream	Name of cream
Rosmarinus Officials L.	Rosemary cream
Farabi base cream	
*Stevastenaryl alcohol*	
*Petroleum gel*	
*Glycerin*	
*Mineral Oil*	
*Preservative*	
*Antioxidants*	
Farabi base cream	Placebo cream
Standard edible brown color	

A pharmacist consulting professor coded 48 tubes of rosemary cream and 48 tubes of placebo cream in the same tubes (code A and B), and the researcher was blinded to the procedure. Both the researcher and the study participants were blinded to the content of the prepared cream as well.

One of the researchers (the second author), who was a master’s student of midwifery with work experience in the delivery department, measured the length and depth of the episiotomy incision after the delivery using a sterile swap and graded ruler after wearing sterile gloves and masks and examined the perineum in terms of episiotomy extension or rupture in other areas. Afterward, the perineum was repaired using 0 and 2-0 chromic catgut suture. After the childbirth, personal information and labor information forms were completed for the participants and they were assigned into groups of intervention and control, according to the previous random block allocation. Participants had similar conditions in terms of the interventional factors, such as the type of episiotomy (mediolateral), repair method, type of suture, and the amount of anesthesia used before incision and repair.

In addition to routine treatment, cream packages (rosemary and placebo) were distributed to the intervention and control groups by the researcher who was blinded to their content. The mothers applied the first dose of the cream in the first 12 h postpartum (at least 2 h after episiotomy repair) in the hospital under the surveillance of the researcher. The mothers in both groups were asked to wash their hands and perineum with normal saline, dry the area, apply the rosemary cream or placebo cream (in a dose of 2 cm) on the sutured area, and use the cleansed sanitary pad after 1-2 min. They were required to apply the cream twice a day for 10 consecutive days (days 1 to 10) postpartum.

Furthermore, the researcher presented educational booklets (containing information on the care of perineum and sutures, personal health, nutrition, amount of physical activity, cream usage, the fourth and tenth-day follow-ups reminder, the place of referral, and the telephone number of the researcher) to both groups.

Based on the hospital routine, cephalexin capsules (500 mg) every 6 h for 7 days were prescribed for all mothers at discharge. Mefenamic acid capsules (250 mg) every 8 h were prescribed for patients who complained of pain. The number of times of taking analgesia, antibiotic and rosemary cream was recorded by the mother in the daily form of drug use registration.

The mothers were requested to contact the researcher (if necessary) in the event of fever and shivering, sensitivity to the cream in general or in the wound area, severe pain, swelling, burning sensation, itching, stiffness, dryness, and purulent discharge in the wound area. At the researcher’s discretion, patients could be transferred to the hospital in case of the occurrence of such complications, and they were asked to record complications in the complication registration form. The exclusion criteria were applied at all stages of the study, and the participants who met any of the exclusion criteria were excluded from the study. The participants were asked to discontinue the intervention after the occurrence of any complication and record complications accordingly.

Baseline evaluation was performed to determine the appearance of episiotomy wound in the first 12 hours postpartum and before the intervention. Episiotomy wound healing rate was assessed in lithotomy position by the researcher using REEDA wound measurement scale on days 4 and 10 postpartum. Using this tool, 5 criteria of redness, edema, ecchymosis, discharge and approximation of the wound edges were examined. The lower the total score, the greater the improvement.

### Statistics analysis

Data were analyzed using SPSS software (Version 19) through the Mann-Whitney test, Chi-square test, Fisher’s exact test, and Student’s t-test. The effect size was calculated by Vargha and Delaney method [34]. In this method, the effect size will be small for the range (0.56-0.64) or (0.34-0.44), medium for the range (0.64-0.71) or (0.29-0.34) and large for the range (≥0.71) or (≤0.29). A *p-*value less than 0.05 (*P <* 0.05) was considered statistically significant.

## Results

**Fig. 1:**
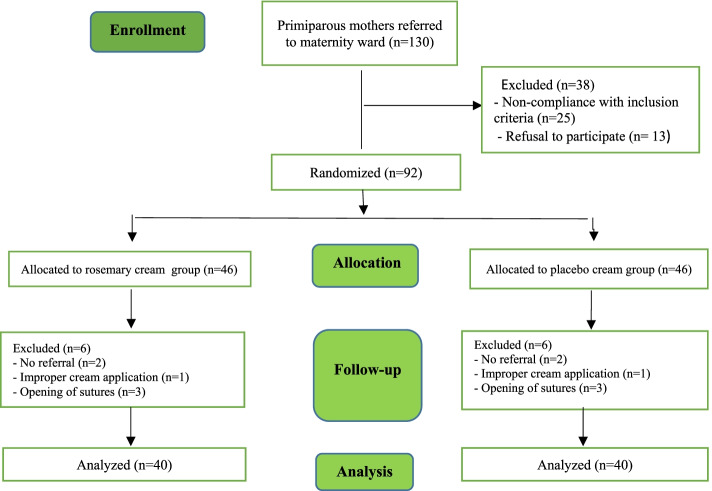
Flowchart of study participants

In this study, 130 individuals were evaluated for eligibility and 92 patients were enrolled in the study. The study participants were then assigned to the rosemary and placebo cream groups. During the study, 12 people were excluded due to lack of referral, improper application of the cream, and ruptured stitches. Eventually, 80 women (40 women in each rosemary and placebo group) were evaluated (Fig. 1).

In terms of education level, 42.5 and 40% of study participants in the groups of intervention and control were high school graduates, respectively. Chi-square test results showed that there was no significant difference between the level of education in the study groups (*P* = 0.86). Regarding the status of employment, the majority of participants (90%) in the intervention group and all participants (100%) in the control group were housewives. There was no significant difference between the status of employment in the two study groups (*P* = 0.11).

There were no statistically significant differences between the two groups in terms of demographic and midwifery characteristics, such as maternal age, body mass index, gestational age, baby weight, baby’s head circumference, episiotomy length, number of episiotomy stitches, and episiotomy repair duration (Table 2).

**Table 2:** Comparison of demographic and midwifery participants in two groups of intervention and control

Variable	Intervention Group Mean ± SD^a^	Control group Mean ± SD	***P-value***^***b***^
Women’s age (Year)	23.55 ± 5.002	25.02 ± 5.05	0. 19*
Body mass index (kg/m2)	23.71 ± 1.22	23.85 ± 1.12	0. 57*
Gestational age (weeks)	39.45 ± 1.15	39.55 ± 1.12	0.68*
Baby weight (g)	2943.83 ± 579.61	3025.75 ± 229.99	0.71**
Neonatal head circumference (cm)	34.77 ± 2.83	34.22 ± 1.24	0.53**
Episiotomy length (cm)	3.85 ± 0.71	3.68 ± 0.79	0.26**
Number of episiotomy sutures	6.27 ± 1.32	5.80 ± 1.47	0.09**
Duration of episiotomy repair (min)	38.12 ± 10.23	37.42 ± 9.01	0.74*

^a^ Standard Deviation

^b^ Significance level: *P* < 0.05

* Independent t-Test

** Mann-Whitney U test

The results of this study showed that before the intervention, the mean scores of REEDA were not significantly different in groups of intervention and control (*P* = 0.68). The mean score of episiotomy wound healing decreased on days 4 and 10 post-intervention in both intervention and control groups; however, the decrease was higher in the group of intervention. Despite this difference, the mean score of episiotomy wound healing was not significantly different in the intervention and control groups at day 4 post-intervention (*P* = 0.17). Also, on day 10 post-intervention there was a significant difference between the groups of intervention and control in terms of the mean score of episiotomy wound healing (*P* < 0.001) (Table 3).

**Table 3:** Comparison of mean score of the episiotomy wound healing on the days 4 and 10 post-intervention in each study group

Time	Intervention Group Mean ± SD^**a**^	Control group Mean ± SD	***P-value***^***b***^	Effect size^**d**^	CI^**c**^ for effect size
Before intervention (the first 12 hours)	7.42 ± 1.79	6.72 ± 1.58	0.68*	0.62	0.49-0.71
Four days after the intervention	3.82 ± 0.93	4.25 ± 1.29	0.17*	0.41	0.30-0.53
Ten days after the intervention	0.75 ± 0.74	3.32 ± 2.54	< 0.0001*	0.11	0.05-0.19

^a^ Standard Deviation

^b^ Significance level: *P* < 0.05

^c^confidence interval

^d^ The effect size is small for the range (0.56-0.64) or (0.34-0.44), medium for the range (0.64-0.71) or (0.29-0.34) and large for the range (≥0.71) or (≤0.29)

* Mann-Whitney U test

Evaluation of wound healing variables (i.e., redness, edema, ecchymosis, discharge, and approximation of the wound edges) on day four post-intervention showed no significant difference in the mean scores of redness (*P* = 0.77), edema (*P* = 0.29), ecchymosis (*P* = 0.31), and the approximation of the wound edges (*P* = 0.64) between the two study groups. However, there was a statistically significant difference between the two study groups in terms of the wound discharge scores (*P* < 0.001).

The two study groups were compared in terms of the scores of redness, edema, ecchymosis, wound discharge, and approximation of the wound edges on day 10 after the intervention. The results showed statistically significant differences between the two study groups in terms of the scores of redness (*P* < 0.001), wound discharge (*P* < 0.001), and approximation of wound edges (*P* < 0.001); however, no statistically significant difference was observed between the study groups in terms of the edema score (*P* = 0.39) (Table 4).

**Table 4:** Comparison of the study groups in terms of the mean scores of redness, edema, wound discharge, and approximation of the wound edges, based on the REEDA scale, on days 4 and 10 post-intervention

Variable		Intervention Group Mean ± SD^**a**^	Control group Mean ± SD	***P-value***^***b***^
Redness	Four days after intervention	0.7 ± 0.51	0.67 ± 0.57	0.77*
	Ten days after intervention	0	0/5 ± 0/93	0.001*
Edema	Four days after intervention	0.82 ± 0.44	0.72 ± 0.71	0.29*
	Ten days after intervention	0.05 ± 0.22	0.10 ± 0.30	0.39*
Ecchymosis	Four days after intervention	0	0.02 ± 0.15	0.31*
	Ten days after intervention	0	0	-------c
Discharge	Four days after intervention	1.32 ± 0.57	1.8 ± 0.51	< 0.001*
	Ten days after intervention	0.30 ± 0.46	1.35 ± 1.14	< 0.001*
Approximation of the wound edges	Four days after intervention	0.97 ± 0.27	1.02 ± 0.35	0.64*
	Ten days after intervention	0.35 ± 0.48	1.37 ± 0.74	< 0.001*

^a^ Standard deviation

^b^ Significance level: *P* < 0.05

^c^ The sample size in the desired variable is not large enough to perform the test

* Mann-Whitney U test

Most participants in the two study groups took the prescribed antibiotics regularly. Therefore, there was no significant difference between the two groups in terms of analgesic and antibiotic use (*P* > 0.05) [Table 5].

**Table 5:** Comparison of the number of days of analgesic and antibiotic use during the first 10 days post-partum in the two study groups

Time	InterventionGroup Mean ± SD^**a**^	Control group Mean ± SD	***P-value***^***b***^
Number of days of analgesic use	6.81 ± 3.20	6.74 ± 3.18	0. 50*
Numberof days of irregular antibiotic use	2.05 ± 0.42	2.08 ± 0.43	0. 75*

^a^ Standard deviation

^b^ Significance level: *P* < 0.05

* Independent t-Test

No complication caused by rosemary and placebo cream application was reported by mothers in both study groups.

## Discussion

This study aimed to determine the effect of topical application of rosemary cream on episiotomy wound healing. The results of this study showed that the rate of wound healing was higher in the group of intervention, compared to controls on day 10 post-intervention, and the difference was significant between the two study groups. Moreover, the mean score of wound healing (based on the REEDA score) on day 10 post-intervention was approximately 4.4 times lower in the intervention group, compared to the control group, indicating the decreased duration of wound recovery in the group of rosemary cream, compared to the placebo group. In addition, the mean score of wound healing in the control group on day 10 postpartum was close to that in the group of rosemary cream on day 4 postpartum. Therefore, based on the obtained results, rosemary cream accelerated the episiotomy wound healing by 6 days in the study group compared to the control group. The effect of rosemary cream on wound healing was more pronounced from day 4 to day 10 postpartum. This improvement in wound healing by rosemary cream was mainly associated with better scores obtained for redness, wound discharge, and approximation of the wound edges.

In this study, the significant difference between the two groups in terms of wound healing can be attributed to the aforementioned rosemary compounds and properties.

Rosemary prevents leukocyte migration in the body, which in turn reduces the number of leukocytes (white blood cells) at the inflammation site and leads to an anti-inflammatory reaction. Rosemary also inhibits inflammatory agents or substances that enhance or exacerbate inflammation, such as nitric oxide and inflammation-related genes. Rosemary also has strong antioxidant properties that reduce oxidative stress and the accumulation of free radicals in the body. The antioxidant activity of rosemary is attributed to its chemical compounds. Phenolic diterpenes, such as carnosic acid, carnosol, and rosmarinic acid are identified as the most effective antioxidants in rosemary. The antimicrobial properties of rosemary also depend on its chemical composition, which can be very different based on location, water, and harvest time [35].

The results of the study conducted by Christopoulou et al. (2021) showed that rosemary extract had antiviral, antifungal, antibacterial, and antioxidant properties [36]. The results of the study conducted by Khoshoei et al. (2022) showed that the healing rate of pressure ulcers was higher in patients admitted to ICU in the intervention group 1 week after the application of rosemary ointment, compared to the control group [26]. Mouthwash containing rosemary extract healed oral mucosal wounds in the study performed by Bestango in 2017 [25]. The study by Dehghani et al. (2019) showed that rosemary extract could prevent throat ulcers due to tracheal intubation [37].

Based on the evidence, rosemary compounds prevented the in vitro formation of inflammatory intermediates, such as prostaglandins, leukotrienes, and cytokines [26].

The wound healing stages were evaluated using the REEDA scale through the evaluation of five healing factors, including redness, edema, ecchymosis, discharge, and approximation of the wound edges [31]. The evaluation of these five factors in the present study revealed significant differences between the study groups in terms of redness on day 10, wound discharge on days 4 and 10, and the approximation of the wound edges on day 10 postpartum. Although the mean score of edema on day 10 postpartum in the intervention group was lower than the control group, no significant difference was reported between the two study groups, which could be due to the small sample size.

The obtained results indicated that rosemary cream reduced redness, wound discharge, and distance between the episiotomy wound edges. Therefore, in this study, the improvement of redness and wound discharge in the episiotomy site in the group of intervention can be attributed to the anti-inflammatory and antioxidant effects of rosemary compounds [35].

In the study performed by Labib et al. (2022), improvement in wound contraction and increased re-epithelialization were observed in the groups treated with rosemary essential oils [23]. The results of the studies conducted by Nejat et al. (2014, 2015) and Farahpour et al. (2017) revealed a significant difference in wound inflammation in rats treated with the ointment containing rosemary essential oil (3%) on days 4 and 8 post-intervention, compared to the control group. In addition, an increase was observed in the formation of new blood vessels, migration of fibroblasts to the wound site, epithelium regeneration, collagen synthesis and secretion, and increase in epithelial tissue thickness in the rosemary ointment group, which resulted in an acceleration in the wound healing process. Therefore, the healing was accelerated in the second stage of wound healing in the wounds treated with rosemary essence [24, 38, 39].

The results of the study performed by Izadpanah and Rahmanpour (2017) showed that the application of rosemary essence led to a significant increase in the percentage of recovery, the number of macrophages, fibroblasts, blood vessels, and a decrease in the number of neutrophils in the wound area of the rats’ skin, compared to the control group [40]. The study conducted by Araujo et al. (2017) aimed to show the effect of rosemary essence on the improvement of skin complications in rats in Brazil. The results revealed that rosemary essence decreased inflammation and increased wound contraction, epithelialization, reconstruction of epithelial tissue, angiogenesis, and collagen deposit in the treated wounds, indicating the therapeutic potential of the rosemary plant for topical applications [27]. These findings were consistent with the results obtained in the present study regarding the decrease in the redness and distance between wound edges on days 4 and 10 postpartum in the intervention group and acceleration of episiotomy wound healing on day 10 postpartum.

The strength of the present study includes the evaluation of the episiotomy wound recovery using the REEDA scale; controlling interventional variables; training and evaluation by the same researcher for both groups; performing episiotomy and suturing by trained midwives according to the standard protocol; using identical episiotomy and repair method, suture type, and amount of anesthesia for everyone.

Regarding the limitations of the present study, one can refer to the impossibility of controlling all factors affecting wound healing, such as nutritional status, the level of physical activity of the participants, and the dissimilarity of participants’ immune systems. These limitations were partially overcome by the provision of similar training to participants and the adoption of a randomized sampling strategy. Another limitation regarding different pain thresholds in different individuals was also partially resolved by random sampling of the participants.

## Conclusion

To the best of our knowledge, this has been the first human study on the effect of rosemary cream on episiotomy wound healing. The results of this study suggested that rosemary cream can be an effective and safe treatment for episiotomy wound healing. Eventually, further supplementary studies with a similar methodology and a larger sample size should be conducted to confirm the findings of this study.

## Data Availability

We do not have a link to the data, and the data is in an SPSS file. If necessary, the datasets used and analyzed in the current study are available on request from the corresponding author upon reasonable request.

## Abbreviations

REEDA: Redness, Edema, Ecchymosis, Discharge, Approximation of the wound edges
CONSORT: Consolidated Standards of Reporting Trial
